# Operability of atrial septal defect with borderline pulmonary vascular resistance index: A study in developing country

**DOI:** 10.3389/fsurg.2022.1031451

**Published:** 2022-10-20

**Authors:** Oktavia Lilyasari, Rini Istisakinah, Rina Ariani, Budi Rahmat, Lies Dina Liastuti, Yovi Kurniawati, Hary Sakti Muliawan, Renan Sukmawan

**Affiliations:** ^1^ Department of Cardiology and Vascular Medicine, Faculty of Medicine, Universitas Indonesia, National Cardiovascular Center Harapan Kita, Jakarta, Indonesia; ^2^ Thoracic, Cardiac, and Vascular Surgery Division, Department of Surgery, Faculty of Medicine, Universitas Indonesia - Pediatric and Congenital Heart Surgery Unit, Department of Surgery, National Cardiovascular Center Harapan Kita, Jakarta, Indonesia

**Keywords:** atrial septal defect (ASD), pulmonary hypertension (PAH), pulmonary vascular resistance index (PVRI), surgical closure of ASD, mortality, morbidity, cardiac catherization

## Abstract

**Background:**

Pulmonary arterial hypertension secondary to atrial septal defect (ASD) is an important determinant of morbidity and mortality in defect closure. We aimed to compare perioperative outcome between preoperative borderline and low pulmonary vascular resistance index (≥4 WU.m^2^ and <4 WU.m^2^, respectively) in surgical closure of secundum atrial septal defect with concomitant pulmonary arterial hypertension.

**Methods and results:**

This was a single-center retrospective cohort study between January 2015 and January 2020. We classified patients with low and borderline PVRI who underwent ASD closure and recorded the perioperative outcomes.

**Results:**

We analyzed a total of 183 patients with atrial septal defect and pulmonary arterial hypertension; 92 patients with borderline PVRI and 91 patients with low PVRI. Borderline pulmonary vascular resistance index was not associated with increased risk of postoperative mortality (*p* = 0.621; OR0.48, 95% CI 0.04–5.48), but associated with higher risk of overall morbidity in bivariate analysis (*p* = 0.002; OR3.28, 95% CI 1.5–6.72). Multivariate analysis showed positive association of borderline pulmonary vascular resistance index (*p* = 0.045; OR2.63, 95% CI 1.02–6.77) and preoperative tricuspid valve gradient ≥64 mmHg (*p* = 0.034; OR2.77, 95% CI 1.08–7.13) with overall morbidity.

**Conclusion:**

There is no difference in incidence of in-hospital mortality between preoperative borderline and low pulmonary vascular resistance index patients. However, preoperative borderline pulmonary vascular resistance index and tricuspid valve gradient ≥64 mmHg are associated with increased overall morbidity after surgical closure in secundum atrial septal defect patients with pulmonary arterial hypertension.

## Introduction

Atrial septal defect (ASD) is one of the most common congenital heart diseases (CHD) found with secundum ASD as the majority type. Sixty-nine percent of adult congenital heart disease (ACHD) patients in Indonesian National Cardiac Center Harapan Kita (NCCHK) are ASD patients. Due to its natural course, secundum ASD patients were often asymptomatic and diagnosed late in adulthood (1). Moreover, developing countries unexceptionally Indonesia, have their own local barriers -geographic, economic, human resources, scarcity of high-end equipment- in providing optimal medical care for such patients (2). Therefore, patients often came as “late presenters” of whom already have challenging courses.

Persistent interatrial shunt in uncorrected ASD leads to development of pulmonary arterial hypertension (PAH), and later pulmonary vascular disease (PVD). This irreversible condition denotes significant changes in pulmonary vascularization and attempt on defect closure would be hazardous (1, 3). Closure of the defect should have taken place before any irreversible changes occurred, preferably when pulmonary hypertension (PH) has not developed. Previous study showed surgical ASD closure has a favorable outcome with low mortality rate (<3%) (1). Unfortunately, preoperative PAH secondary to congenital heart disease is an important determinant for perioperative morbidity and mortality as well as long-term postoperative survival (4). Presence of PAH could lead to incidence of postoperative pulmonary hypertension crisis (PHC) that has disastrous effect with mortality rate as high as 20% (5). Given the life-threatening complications associated with pulmonary hypertensive crisis, stratifying the risk of preoperative PAH would determine the outcome after surgery.

Existing guideline stated, CHD with systemic-to-pulmonary shunts with preoperative pulmonary vascular resistance index (PVRI) < 4 WU.m^2^, considered as low PVRI, is appropriately safe to close. However, preoperative PVRI ≥4 WU.m^2^ deemed to be on grey/borderline zone where development of PVD might already occurred. Meanwhile, patients with PVRI >8 WU.m^2^ -even after acute vasoreactivity test- is thought to have irreversible pulmonary vascular changes and will not have favorable outcomes (6). Patients with dubious PVRI value still have the chance for defect correction with noteworthy consideration where individual patient evaluation should be performed in tertiary centers. Some of those patients received pulmonary vasodilator therapy to achieved operability criteria (treat-to-repair concept) (6, 7). Studies regarding this challenging group are still scarce, mostly are case reports with various kind of systemic-to-pulmonary shunt defect. None of the studies have showed short or long-term outcome after defect closure in borderline PVRI especially in secundum ASD patients, since these patients in developing countries tend to come as late presenter and often had develop pulmonary hypertension. The study aimed to investigate the impact of preoperative borderline PVRI compared to low PVRI on short-term outcome after surgical ASD closure in secundum ASD patient with PAH.

## Materials and methods

### Study designs and participants

This was a single-center and a retrospective observational study comparing the perioperative outcome of preoperative borderline and low PVRI groups after surgical closure of secundum ASD. We identified adult (≥18 years old) patients with secundum ASD with PAH (ASD-PAH) who underwent surgical closure in our hospital between January 2015 and January 2020. Patients with ASD with other concomitant CHD, patients who underwent surgical procedures other than ASD closure and/or valve repair/replacement were excluded. We calculated the sample size for this particular study and for the 90% of statistical power, the minimal size would be 87 patients per group or 174 patients in total. The study was reviewed and approved by the Ethical Committee of Department of Cardiology and Vascular Medicine, Faculty of Medicine Universitas Indonesia, National Cardiovascular Center Harapan Kita (Ethical Approval Number: LB.02.01/VII/429/KEP.029/2020).

### Data collection

We collected demographic, clinical, electrocardiographic, echocardiographic, RHC, surgical and post-surgical data from medical records. Age were categorized into ≥40 and <40 year old (8). Secundum ASD was mainly diagnosed transthoracic echocardiography (TTE) and trans-esophageal echocardiography (TEE). Data of preoperative defect size was obtained with TEE when available and confirmed during procedure. The size of left ventricle (LV) was determined by end diastolic diameter (EDD) index and considered to be “small-sized” if less than 2.2 cm/m^2^ for male and less than 2.3 cm/m^2^ for female. Decrease right ventricle function defined as tricuspid annular plane systolic excursion (TAPSE) < 17 mm (9).

### Catheterization

PAH was defined for the purpose of this study as mean pulmonary arterial pressure (mPAP) > 20 mmHg, pulmonary capillary wedge pressure < 15 mmHg, and PVR > 3 WU at rest right heart catheterization (RHC) (10). Hemodynamic parameters (Qp/Qs, PVRI, resistance ratio) were calculated using Fick method. Oxygen consumption was estimated with table from La Farge and Miettinen (11). Acute vasoreactivity test (AVT) was carried out with delivery of 100% oxygen (FiO2) *via* face mask for 10 min. Using data from RHC reports, all patients were recalculated for PVRI with WU.m^2^ as the agreed unit (6). This measure was taken due to diversified units that had been used in the past (WU or WU.m^2^ or WU.m-2). Borderline PVRI was defined as pulmonary vascular resistance index ≥4 WU.m^2^ before AVT. Low PVRI was defined as pulmonary vascular resistance index <4 WU.m^2^ before AVT.

### Surgical closure

Referring to our hospital clinical practice guideline, secundum ASD-PAH patients with PVRI < 8 WU.m2 before or after acute vasoreactivity test (AVT) were deemed operable. Surgical data were obtained from the surgical note. Based on previous study, aortic cross clamp (Aox) time will be classified to > 45 min and < 45 min (12). Prolonged cardiopulmonary bypass (CPB) time was defined as CPB time > 100 min (13).

### Endpoint

Primary clinical endpoint was in-hospital mortality after surgery, defined as all-cause mortality events during post-operative period. Secondary clinical end-point was overall morbidity that includes any of pulmonary hypertension crisis event, prolonged intensive care unit stay, and/or mechanical ventilation use. Pulmonary hypertension crisis was defined as acute increase in pulmonary arterial pressure to systemic arterial pressure ratio >0.75 which was usually accompanied by an increase in central venous pressure (>20%), a decrease in blood pressure (>20%) and a decrease in oxygen saturation levels to less than 90% with signs of decreased cardiac output (5). Prolonged intensive care unit (ICU) stay was defined as period stay at ICU for more than two days (14). Twenty-four hour was used as a cutoff point to consider prolonged mechanical ventilation use (15).

### Statistical analysis

Statistical analysis was performed using SPSS software for Macintosh, version 22 (IBM, New York). Categorical variables stated in frequencies and proportions. Continuous variables were expressed as means + standard deviation or medians with ranges when appropriate. For numeric variables, the differences between the two groups were analyzed with *T*-test or Mann-Whitney test, as appropriate. For discrete variable, the differences between two groups were analyzed with the *χ*2 or Fisher's exact test, as appropriate. Certain variables (age, PVRI, size defect, TAPSE, mPAP, TVG, PaO2, CPB time, AoX time) will be categorized according to agreed terms. Variables with *p* < 0.25 in bivariate analysis will be included in multivariate analysis using logistic regression. A two-tailed *p*-value of <0.05 was considered statistically significant.

## Results

### Study population

**Figure 1:**
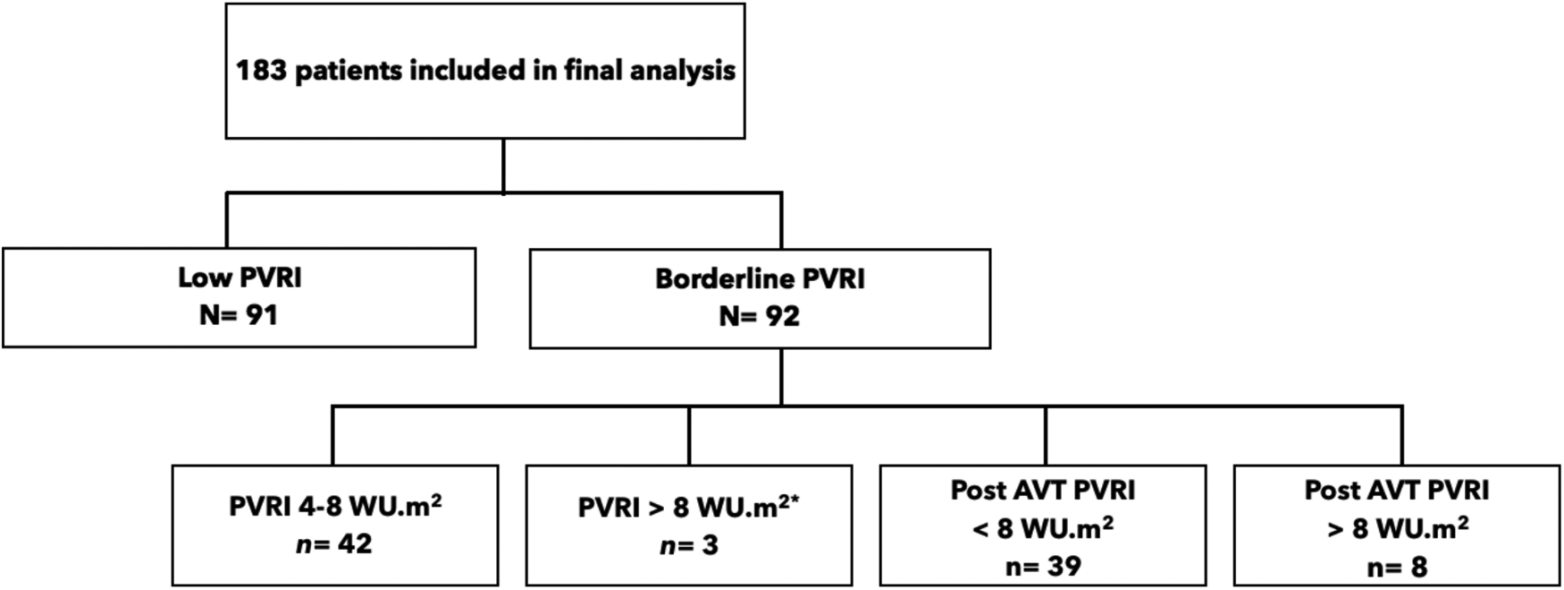
Schematic review of study sample.

During the five-year study period, surgical closure of ASD was attempted in 510 adult patients. Approximately 255 patients met the inclusion and exclusion criteria, however only 183 patients had available complete data and included in final analysis. The median preoperative PVRI was 4.3 WU.m^2^ (0.4–13.5 WU.m^2^). Ninety-two patients (51%) were classified as borderline PVRI group and 91 patients (49%) belonged to low PVRI group. After recalculation there were eleven patients with preoperative PVRI >8 WU.m^2^. Detailed characteristic was summarized in Figure 1.

### Preoperative baseline characteristics

Most of the patients were female (86.3%) with median age of 37 (18–64) years old. More than half of the population preoperative NYHA Fc II (72.7%). We found that atrial fibrillation was present in 26/183 patients (14.2%). Comparison of baseline clinical, echocardiographic and cardiac catheterization between two groups are summarized in Tables 1, 2, respectively. A distinct baseline clinical characteristic existed between two groups except for median age, gender distribution, and comorbidities. However, as variable age was dichotomized into two groups (< 40 and ≥40 years old), low PVRI group had greater proportion of patients with age ≥40 years old (48.4% vs. 32.6%; *p* = 0.03). Preoperative oxygen saturation (SaO_2_) and preoperative partial pressure of oxygen (PaO_2_) in borderline PVRI group were significantly lower compared to low PVRI group (*p* < 0.001). Phospdiesterease-5 inhibitor (Sildenafil) was the drug of choice for preoperative pulmonary vasodilator treatment (80.7% vs. Beraprost 13.8%). Combination therapy were used in 5.5% patients. Comparison between groups showed a contrasting echocardiography and cardiac catheterization features. Through basic echocardiography it showed that borderline PVRI group had significantly decreased RV function (TAPSE) with preoperative tricuspid valve gradient (TVG) that was higher than those of low PVRI group. However, there was no significant difference in defect size, LV size, and proportions of mitral regurgitation severity (*p* > 0.05). No difference found in the type of anesthesia used during RHC procedure.

**Table 1:** Baseline characteristic of 183 secundum ASD patients who underwent surgical closure.

Variable	Borderline PVRI (*n *= 92)	Low PVRI (*n *= 91)	*p* value
Age	35 [19–58]	39 [18–64]	0.078
Sex			
Female (*n *= 153)	79 (85.9%)	74 (81.3%)	0.406
Male (*n *= 30)	13 (14.1%)	17 (18.7%)	
NYHA functional class			
NYHA I	11 (12%)	29 (31.9%)	0.004
NYHA II	76 (82.6%)	57 (62.6%)	
NYHA III	5 (5.4%)	5 (5.5%)	
Atrial fibrillation	8 (13.1%)	18 (19.8%)	0.032
Preoperative SaO_2_ (%)	97 (84–100)	98 (92–100)	<0.001
**Comorbidities**			
Previous pulmonary disease	4 (4.3%)	2 (2.2%)	0.518
CTEPH	2 (2.2%)	1 (1.1%)	
Diabetes mellitus	1 (1.1%)	1 (1.1%)	
Hypertension	3 (3.3%)	4 (4.4%)	
History of stroke	0 (0%)	1 (1.1%)	
Thyroid disease	3 (3.3%)	1 (1.1%)	
Preoperative PaO_2_ (mmHg. *n *= 159)	86.7 (55.8–181)	101.4 (71.3–156.7)	<0.001
Preoperative pulmonary vasodilator drugs	70 (76.1%)	39 (42.9%)	<0.001

CTEPH, chronic thromboembolic pulmonary hypertension; NYHA, New York Heart Association; PaO_2_, Oxygen partial pressure; SaO_2_, peripheral oxygen saturation.

**Table 2:** Preoperative diagnostic characteristics by PVRI group.

Variable	Borderline PVRI (*n *= 92)	Low PVRI (*n *= 91)	*p* value
**Baseline Echocardiography**			
Defect size (mm)	32.5 (13–56)	34 (13–80)	0.127
Bidirectional shunt	44 (47.8%)	24 (26.4%)	0.003
LV size (*n* = 173)			
Small-sized LV	21 (24.4%)	29 (33.5%)	0.196
TAPSE (mm. *n *= 178)	22 (11–37)	28.4 (11–41)	< 0.001
Tricuspid regurgitation			
Mild	33 (35.9%)	44 (48.4%)	0.036
Moderate	26 (28.3%)	27 (29.7%)	
Severe	30 (32.6%)	18 (19.8%)	
TVG (mmHg)	75.5 (24–166)	53 (24–110)	< 0.001
Mitral regurgitation			
Mild	37 (40.25)	34 (37.4%)	0.488
Moderate	12 (13%)	12 (13.2%)	
Severe	5 (5.4%)	1 (1.1%)	
Right Heart Catheterization			
Flow ratio	2.6 (1.08–21.34)	3.4 (1.25–25.2)	0.002
mPAP (mmHg)	48 (24–84)	29 (21–75)	< 0.001
Procedural anaesthesia			
Local (*n = *162)	85 (92.4%)	77 (84.6%)	0.099
General (*n *= 21)	7 (7.6%)	14 (15.4%)	

LV, left ventricle; mPAP, mean pulmonary arterial pressure; TAPSE, tricuspid annular plane systolic excursion; TVG, tricuspid valve gradient.

### Surgical procedure and perioperative ICU care

Table 3 summarized surgical procedure and perioperative ICU care between two groups. Median defect size was 30 (15–60 mm) without significant difference between two groups (*p* = 0.376). Most defects were closed with pericardial patch (89.6%), the rest with direct closure and in some cases a small fenestration was created in the surgical patch. Different characteristics found regarding perioperative care. Patients in borderline PVRI group received significantly more supportive drugs (inotropic, vasopressor, and/or inodilator agent) compared to low PVRI group (*p* < 0.001). Similar result was recorded for perioperative pulmonary vasodilator treatment usage. Nitric oxide inhalation was used for two patients as pulmonary hypertension crisis (PHC) treatment. Inhaled Illoprost was given for 50% patients who received perioperative pulmonary vasodilator treatment. Sildenafil was given as substitute for Illoprost or as a single therapy. Median length of stay in the ICU was 1 (1–23) day, while duration of mechanical ventilation was 12 (0–548) hours. Two patients had early extubation in the operating room.

**Table 3:** Surgical procedures and ICU care in 183 secundum ASD patients underwent surgical closure.

Variable	Borderline PVRI (*n *= 92)	Low PVRI (*n *= 91)	*p* value
Surgical closure			
Pericardial patch	84 (91.3)	80 (87.9%)	0.451
Direct closure	8 (8.7%)	11 (12.1%)	
Tricuspid valve repair	53 (57.6%)	41 (45.1%)	0.089
Mitral Valve Repair/Replace	20 (21.7%)	16 (17.6%)	0.479
Aox time (minutes. *n *= 181)	34.5 (12–174)	32 (7–131)	0.281
CPB time (minutes. *n *= 178)	68.5 (28–236)	69.5 (26–221)	0.349
Number of support agent			
Without	3 (3.3%)	13 (14.3%)	< 0.001
1	14 (15.2%)	31 (34.1%)	
2	37 (40.2%)	36 (39.6%)	
≥3	38 (41.3%)	11 (12.1%)	
Perioperative pulmonary vasodilator	44 (47.8%)	8 (8.8%)	< 0.001

Aox, aortic cross clamp; CPB, cardio-pulmonary bypass.

The most common perioperative complication was arrhythmias (15.3%) with new-onset atrial fibrillation as the most frequently encountered. Other complications were stroke (1.6%), pneumonia (16.5%), low cardiac output syndrome (2.7%), and acute kidney injury (3.2%). One of three patients who developed strokes had subarachnoid hemorrhage (SAH). Redo procedures were performed in three patients; two patients due to surgical bleeding and one patient had side port sutured to his superior vena cava.

### Primary endpoint

There was no significant difference in incidence of in-hospital mortality between borderline and low PVRI groups (1.1% vs. 2.2%, OR 0.48 95% CI 0.04–5.48; *p* = 0.621). None of other variables (age, sex, defect size, TAPSE, small-sized LV, TVG, mPAP, PaO_2_, preoperative pulmonary vasodilator, Aox time and CPB time) showed any significant difference, thus multivariate analysis by logistic regression was not performed. Overall incidence of in-hospital mortality was 1.6% (3/183), none of them had pulmonary hypertension-related cause of death. One patient who belonged in borderline PVRI group died from excessive bleeding resulting in uncorrectable hemodynamic state despite optimal effort. Two patients from low PVRI group died from massive non-hemorrhagic stroke and sepsis with multi-organ dysfunction.

### Secondary endpoint

Table 4 detailed the incidence of overall morbidity and each of the components considered. Pulmonary hypertension crisis (PHC) occurred in seven patients and all of them belonged to borderline PVRI group. Five patients had post-AVT PVRI >8 WU.m^2^, after recalculation. The incidence of overall morbidity was higher in borderline PVRI group compared to low PVRI group before adjustment (32.6% vs. 13.2%, OR 3.28 (95% CI 1.5–6.72; *p* = 0.002)) and after adjustment (OR 2.63 (95% CI 1.02–6.77; *p* = 0.045)). Summarized in Table 5, preoperative TVG ≥64 mmHg, mPAP ≥36 mmHg, arterial PaO_2_ < 80 mmHg, and use of pulmonary vasodilator drug preoperatively were predictors for overall morbidity. However, after adjustment, only preoperative TVG ≥64 mmHg remained as independent predictors of overall morbidity (*p* = 0.034). Statistical analysis for overall morbidity summarized in Tables 5, 6.

**Table 4:** Postoperative in-hospital mortality and morbidity.

Postoperative complication	Value (*n *= 183)	*p* value
Mortality	3 (1.6%)	0.621
Morbidity (*n *= 42)		0.002; 0.045^a^
Pulmonary hypertension crisis	7 (3.8%)	
Prolonged ICU stay	33 (18%)	
Prolonged mechanical ventilation	34 (18.5%)	

ICU: intensive care unit.

^a^ adjusted.

**Table 5:** Univariate and bivariate analysis factors influencing overall morbidity.

Variable	Overall morbidity (*n *= 42)	No morbidity (*n *= 141)	*p* value
Borderline PVRI	30 (32.6%)	62 (67.4%)	0.002
Age ≥ 40 years old	15 (20.3%)	59 (79.7%)	0.477
Sex			
Female	35 (22.9%)	118 (77.1%)	0.957
Male	7 (23.3%)	23 (76.7%)	
Defect size ≥ 30 mm	32 (21.8%)	115 (78.2%)	0.442
TAPSE < 17 mm	3 (15.8%)	16 (84.2%)	0.57
TVG ≥ 64 mmHg	33 (34.7%)	62 (65.3%)	<0.001
*Small-sized* LV	11 (22%)	39 (78%)	0.823
mPAP ≥ 36 mmHg	30 (31.9%)	64 (68.1%)	0.003
PaO_2_ < 80 mmHg	9 (37.5%)	15 (62.5%)	0.021
Preoperative pulmonary vasodilator	34 (31.2%)	75 (68.8%)	0.001
Aox time >45 min	12 (22.2%)	42 (77.8%)	0.797
CPB time ≥ 100 min	8 (25.8%)	23 (74.2%)	0.750

Aox, aortic cross clamp; CPB, cardio-pulmonary bypass; LV, left ventricle; mPAP, mean pulmonary arterial pressure; PaO_2_, partial oxygen pressure; PVRI, pulmonary vascular resistance index; TAPSE, tricuspid annular plane systolic excursion; TVG, tricuspid valve gradient.

**Table 6:** Univariate and multivariate analysis of factors affecting overall morbidity after surgical ASD closure.

Variable	COR (95% CI)	*p* value	AOR (95% CI)	*p* value
Borderline PVRI	2.38 (1.31–4.30)	0.004	2.63 (1.02–6.77)	0.045
TVG ≥ 64 mmHg	2.99 (1.64–5.46)	<0.001	2.77 (1.08–7.13)	0.034
mPAP ≥ 36 mmHg	2.72 (1.49–4.95)	0.001	1.55 (0.51–4.67)	0.435
PaO_2_^ ^< 80 mmHg	3.05 (1.15–8.15)	0.026	1.50 (0.53–4.23)	0.439
Preoperative pulmonary vasodilator	2.09 (1.14–3.80)	0.016	1.94 (0.73–5.15)	0.182

CI, confidence interval; mPAP, mean pulmonary arterial pressure; PaO_2_, partial oxygen pressure; PVRI, pulmonary vascular resistance index; TVG, tricuspid valve gradient.

## Discussion

Prevalence of ASD-PAH ranged between 8%–10% (3). However, we reported a higher proportion of PAH in secundum ASD patients who underwent surgical closure (44%; 225/510 patients) in this study. This higher number might be due to our hospital is the national referral center for CHD in which most patients were referred with advanced stage of disease of whom required more specialized intervention. The demographic characteristics in our study were similar with previous studies which were mostly female patient and in third-to-fourth decade (1, 14). Clinical and diagnostic characteristics supported distinct hemodynamic properties between groups whom similar to other studies though no difference in size defect was found between groups. Conflicting findings in other studies exist regarding PH severity and defect size (16, 17). Determination of defect size in available studies was done using echocardiography where the accuracy of the results depends on the view taken, the form of ASD, as well as individual expertise. In our study, measurement of ASD defect size was confirmed at surgery.

Primary endpoint in this study showed no difference in in-hospital mortality between two groups with particularly low mortality rate (1.6%) which concordance with available statement regarding surgical ASD closure safety (1). The causes of death varied but very few had reported PH as the cause. Horvarth et al. (1992) reported two deaths from 166 patients underwent surgical ASD closure with one of them had pulmonary hypertension as the responsible cause of death (18). Although our study shared the same value of in-hospital mortality, we had completely different study subjects compared to other previous studies. All of our subjects had preoperative PH (confirmed by gold standard RHC) which were considered to have higher risk compared to patients without, while in other studies only partial of subjects were accompanied by PH known with different diagnostic approach. Currently, there is no previous study regarding ASD closure similar to ours. Whilst Bando et al. (4) already reported that preoperative PH was independent predictor of mortality after CHD correction [OR 2.2 95% CI 1.05–4.59; *p* = 0.036], three patients who died during the course of our study had no report of pulmonary hypertension crisis and none of the considered variables proven to be the independent cause of mortality.

Discussion about postoperative mortality cannot be separated with the most avoided complication of pulmonary hypertension, pulmonary hypertension crisis. Our study reported similar incidence rate of PHC (3.8%) with the available data (0.75%–4%) (5). High preoperative pulmonary vascular resistance (PVRI >6 WU.m^2^) and less vasoreactivity (decrease PVRI <20% after AVT) are risk factors for postoperative complication in patients with preoperative PH (19). Our study found all patients who experienced postoperative PHC were in the borderline PVRI group with five of them had final PVRI value >8 WU.m^2^ although they had good vasoreactivity.

The risk of mortality in patient experienced PHC is as high as 20% (9). In our study, all of the patients with PHC survived the disastrous event and were fully discharged from hospital in good condition. Furthermore, eleven patients who had preoperative PVRI >8 WU.m^2^ did not experienced mortality and only 6/11 patients had morbidity. These favorable findings might be due to optimal pre-and perioperative care such as administration of optimal preoperative pulmonary vasodilator, good adherence, and practice to recommendation regarding postoperative PH care (sedation, minimal manipulation, acidosis treatment, good hydration, administration of inotropic and inodilator as appropriate, oxygenation, and perioperative pulmonary vasodilator) in our patients (20). These advantages might not available in studies conducted before 1990 though they had lower operability PVRI limit such as in study by Bush et al. (PVRI ≤6 WU.m^2^) and Neutze et al. (PVRI <7 WU.m^2^), where perioperative care not as sophisticated as nowadays and pulmonary vasodilator were not widely used and available (21). Those studies however had CHD type and patient's characteristics that differed from our study. To be of note, there has not been a similar study for secundum ASD-PAH patient either locally or internationally.

Another enticing aspect in our study was more than half of patients (52.2%) in borderline PVRI group did not receive any perioperative pulmonary vasodilator and yet none of them had any event. The decisions to give perioperative therapy was not only based on preoperative hemodynamic but also on postoperative clinical and hemodynamic consideration. Widely known as the principal risk factor for incidence of postoperative PH, high PVRI value is not the solely predictor. Pulmonary vascular reactivity contribute to acute increase of postoperative pulmonary vascular pressure and there were no parameters that can truly predict this (22). Canter et al. found that genetic factor T1450N polymorphism of gene encoding carbamoyl-phosphate synthetase I (an important enzyme for endogenous nitric oxide production) play a role in the increase in pulmonary pressure in postoperative pediatric patients (23). Further research is needed regarding contributing factors.

Expected results for other postoperative morbidity components were reported from our study. Fouly et al. have stated that patients with preoperative PH had longer ICU stay and mechanical ventilation use (24). Patients whom developed postoperative pulmonary hypertension would inevitably had longer duration of mechanical ventilation as part of precautious ICU care and indirectly affect the ICU stay.

We reported borderline PVRI group and preoperative TVG ≥ 64 mmHg as independent predictor for overall morbidity. Different results reported by Horer et al. in which preoperative atrial fibrillation and greater defect size had longer ICU stay (14). However this study had different characteristic regarding PH, only some of them had preoperative PH with mean mPAP 18.6 ± 6.4 mmHg and PVR 2.26 ± 1.67 WU.

To our knowledge, our study was the first to assess the association of preoperative TVG with clinical outcome after surgical closure of ASD associated with PAH. Other studies mainly use systolic pulmonary arterial pressure (sPAP) both for defining PH and as predictor factor. Tricuspid valve gradient is the component for calculating estimated sPAP which represents the interaction between right ventricle and afterload (PVR) (25). Thus, an increase in PVR would be followed with an increase in TVG, which could be seen in patients with overall morbidity had similar proportion for borderline PVRI and TVG ≥64 mmHg groups.

Severe left ventricular dysfunction after ASD closure was a possible complication in adult patients. This condition would lead to low cardiac output syndrome (LCOS) and affect the outcome after closure. This undesired complication was postulated due to small LV size that can cause LV diastolic dysfunction (26). Our center found that LV end diastolic volume index ≤53.3 ml/m^2^ by magnetic resonance imaging (MRI) was predictor of LCOS after surgical ASD closure (27). Not all of our patients in this study were performed preoperative MRI, therefore we use echocardiography parameter to define small-sized LV. No difference in outcome was found regarding this variable.

Although the study was a retrospective study, this study managed to depict our achievements as national referral center for congenital heart disease. Existing barriers did not hinder our effort to provide optimal care in accordance with existing guidelines and evidence-based practice. Our study only remarked the short-term outcome whereas preoperative PAH could affect long-term outcome in relation to PAH reversibility after defect closure which raised another concern. Further study was needed to evaluate the PAH reversibility in ASD patients with preoperative borderline PVRI.

## Conclusion

The result of present study indicates that adult secundum ASD-PAH who had preoperative borderline PVRI did not have any different for in-hospital mortality post-surgical closure compared to low PVRI. However, the borderline PVRI group had higher incidence of overall morbidity. These findings supported the operability of patient with borderline PVRI, yet caution must be made for perioperative care to anticipate possible morbidity. Hence, readiness of the center to overcome post-operative pulmonary complication plays a key role in determining perioperative outcome.

## Data Availability

The original contributions presented in the study are included in the article/Supplementary Material, further inquiries can be directed to the corresponding author/s.
